# Role of Endoscopy in the Diagnosis and Management of Esophageal Cancer

**DOI:** 10.3390/jcm14228169

**Published:** 2025-11-18

**Authors:** Jennifer Ma, Sharon Pan, Rachel Mortan, Faisal Shaukat Ali, Nirav Thosani, Vaibhav Wadhwa

**Affiliations:** 1 McGovern Medical School, University of Texas Health Science Center, Houston, TX 77030, USA; jennifer.ma@uth.tmc.edu (J.M.); sharon.pan@uth.tmc.edu (S.P.); rachel.e.mortan@uth.tmc.edu (R.M.); 2 Section of Gastroenterology, Raymond G Murphy Veterans Affairs Medical Center, Albuquerque, NM 87108, USA; faisalshaukatali@gmail.com; 3 Section of Gastroenterology and Hepatology, Department of Medicine, University of New Mexico, Albuquerque, NM 87106, USA; 4 Center for Interventional Gastroenterology at UTHealth (iGUT), Division of Gastroenterology Hepatology and Nutrition, McGovern Medical School, University of Texas Health Science Center, Houston, TX 77030, USA; nirav.thosani@uth.tmc.edu

**Keywords:** esophageal adenocarcinoma, esophageal squamous cell carcinoma, endoscopy, esophagogastroduodenoscopy

## Abstract

Esophageal cancer cases are predicted to reach 957,000 by 2040. Prior mortality rates average 6.5% in men and 2.2% in women, with a poor 5-year prognosis of 20%. A deficiency in screening guidelines, an incomplete understanding of pathophysiology, and limited treatment options contributed to this poor prognosis. Now, as technology and knowledge evolve, endoscopy serves a primary role in improving morbidity and mortality around esophageal cancer, in which early detection and treatment play a profound role. Advances in diagnostic modalities, including higher frequency ultrasound, acquisition of larger specimens, and nodal characterization, all improve esophageal cancer diagnostic accuracy and treatment planning. This is primarily due to earlier detection of precursor lesions, eradication with complete resection, and more informed surveillance. Prior management with esophagectomy has now evolved to include endoscopic submucosal dissection, mucosal resection, ablation, stent placement, fiducial markers for radiotherapy, sponge vacuum, and more. These endoluminal remedies are curative, palliative, or post-intervention solutions, thereby reducing the surgical risk, morbidity, and mortality associated with esophageal cancer. This review article details the diagnostic and therapeutic role of endoscopy in esophageal cancer.

## 1. Introduction

Over the years, esophageal cancer (EC) has remained among the most common causes of cancer-related death. In 2021, esophageal cancer saw an estimated 576,000 new cases and 538,000 deaths globally, ranking 11th in cancer incidence and 7th in cancer mortality [1]. The global age-standardized incidence rates (ASIR) and mortality rates (ASMR) per 100,000 person-years were 6.65 and 6.25 per 100,000, respectively. By 2050, the ASIR and ASMR are projected to be 6.17 and 5.23 per 100,000, respectively [1]. Overall, esophageal cancer has higher incidence rates in men compared to women at 7.6% vs. 2.6%, respectively, and higher mortality rates at 6.5% vs. 2.2%, respectively. There are two main histological types—esophageal squamous cell carcinoma (ESCC), which is most prevalent worldwide and declining in incidence, and esophageal adenocarcinoma (EAC), with rising incidence in developed countries [2]. Risk factors, screening guidelines, and management vary by type of lesion. The key to early detection of both EAC and ESCC is centered on early detection of the precursors. However, patients often present with advanced disease; in a 2004–2015 database review, 36.9% of patients presented with stage IV EAC and 26.8% stage IV ESCC [3]. Overall, there remains a poor 5-year prognosis for esophageal cancer at about 20% in part due to limited screening guidelines [3]. Previously, the main utility of endoscopy was primarily diagnostic, with better visualization and tissue sampling; however, with technology and knowledge evolving, endoscopy now has a role from beginning to end with screening, surveillance, diagnostics, curative treatment, and palliative treatment. This review article highlights the current diagnostic and therapeutic role of endoscopy in esophageal cancer and future directions.

## 2. Epidemiology

### 2.1. Esophageal Squamous Cell Carcinoma

It is estimated that ESCC incidence in the US is about 1.2 per 100,000, where it is comparatively less common than EAC [4,5]. However, ESCC accounts for most of the esophageal cases globally (approximately 80%) [6]. In the United States, black patients are disproportionately affected by ESCC compared to other groups [7]. Worldwide, ESCC is the most common subtype of esophageal cancer for both male and female patients, with incidence rates of up to 7.8 and 3.2 per 100,000, respectively. ESCC is most common in regions such as Eastern and Southeastern Asia and Southern Africa. In countries such as Malawi, Kenya, Sudan, Pakistan, and Bangladesh, esophageal cancer ranks within the top three causes of death [8].

Major risk factors associated with ESCC include alcohol consumption, tobacco use, as well as frequent consumption of hot beverages, as recently discussed in the literature, given the higher prevalence of ESCC in areas in Asia with common practices of drinking hot tea [5,9]. There is no obvious gender distribution for ESCC; however, African American populations are reported to have higher risks compared to Caucasian populations in the US [5]. East, South, and Central regions of Asia, in addition to East and South Africa, account for the majority of esophageal cancer cases globally, most of which are ESCC [6]. Central America and West Africa have been noted to have the lowest rates of esophageal cancers [6]. A diagnosis of atrophic gastritis can double the risk of ESCC, while achalasia increases the risk of ESCC by 16 times within the first 24 years of diagnosis [10]. Howell–Evans syndrome is due to a mutation in the *RHBDF2* gene and causes an inherited form of tylosis (hyperkeratosis of the palms and soles); this mutation is strongly associated with the development of ESCC [10]. *TP53* mutation is seen in about 92% of ESCCs; other common mutations are in *NOTCH1* and *NOTCH3* (33% and 25%, respectively) [11].

### 2.2. Esophageal Adenocarcinoma

Esophageal adenocarcinoma (EAC) was initially quite rare until the 1960s, when the United States incidence rate began to rise, eventually overtaking ESCC by the 1990s [12]. Conversely, in Africa and Asia, ESCC predominates. This rapid rise in EAC in Western countries was not well understood initially, but possible contributions are hypothesized to be from obesity, gastroesophageal reflux disease (GERD), and a decline in Helicobacter pylori infections. It is now estimated that the incidence of EAC in the US is about 2.7 per 100,000. EAC accounts for around 14% of esophageal cancers worldwide and is most prevalent in developed countries in Northern and Western Europe, Canada, Australia, and the United States. Morgan, Eileen et al. predict 957,000 new esophageal cancer cases in 2040, 143,000 of which will be EAC [8]. If current mortality rates remain stable, there will be an estimated 61.8% increase in the number of deaths from 2020 to 2040, a total of 880,000. If diagnosed early, when isolated to mucosal or submucosal layers, EAC has a favorable prognosis. Most patients who are diagnosed with early-stage EAC have no symptoms and tend to be over the age of 65. Disappointingly, most cases are diagnosed at a later stage when the prognosis is poor. The 5-year survival rate for patients with EAC is comparable to liver, lung, or pancreatic cancer, around 20% [6,13].

Risk factors for EAC include GERD, central obesity, and smoking; and possible EAC-protecting factors include acid suppression, nonsteroidal anti-inflammatory drugs (NSAIDs), and statins [12]. Dietary alcohol does not appear to increase the risk of EAC. The chronic inflammation caused by eosinophilic esophagitis (EOE) raises concerns for malignant transformation, but Syed et al. found no association between EOE and esophageal cancer as a whole [4]. The only known precursor lesion to EAC is Barrett’s esophagus (BE), which is defined by metaplasia of the stratified squamous epithelium in the esophagus with columnar epithelium with goblet cells. The most common gene mutation associated with EAC is *TP53* in 72% of cases, followed by *p16/CDKN2A* in about 12% of cases [11].

### 2.3. Other Rare Esophageal Cancer Types

While EAC and ESCC are the two most common types of esophageal cancer, some other rarer forms of EC are subtypes like small-cell carcinoma, lymphoma, melanoma, and gastrointestinal stromal tumor (GIST) [14]. Small-cell carcinoma of the esophagus accounts for 0.8 to 2.8% of all diagnosed esophageal cancers, and the literature reports around 600 known cases [14]. The survival rate of small-cell carcinoma of the esophagus is dismal, with a median survival of only 8–13 months, and most deaths occur within 2 years [15]. Esophageal lymphoma accounts for less than 1% of all esophageal cancer diagnoses, with less than 30 cases reported in the literature [14]. Human immunodeficiency virus is associated with esophageal lymphoma and is considered an AIDS-defining illness and a marker of poor prognosis [14]. The average survival recorded by Saddoughi et al. was approximately 67 months after surgical resection. Primary malignant melanoma of the esophagus is an aggressive tumor that is usually localized to the distal esophagus with no known predisposing factors [16]. It accounts for about 0.1–0.2% of reported esophageal cancers and has less than 400 cases reported [14,16]. The five-year survival rate has a wide range from 2.2 to 37.5% [14]. Esophageal GISTs also account for less than 1% of all esophageal cancers, have a higher mitotic rate, larger tumor size, and have a worse prognosis than gastric GISTs [14,17]. Esophageal carcinosarcoma accounts for 0.5–2.8% of esophageal cancers [18,19]. Chen et al. report a three- and five-year survival rate in a group of 24 patients as 83.3% and 70.8%, respectively [18]. Lastly, esophageal leiomyosarcoma is a rare cancer that accounts for less than 1% of all diagnosed esophageal cancers [20]. These tumors are relatively slow growing, and surgical resection is the treatment of choice [21]. The three- and five-year survival rates are 42.8% and 32.1%, respectively [21].

## 3. Screening and Surveillance

### 3.1. Barrett’s Esophagus

The role of endoscopy in screening for esophageal adenocarcinoma (EAC) is based on Barrett’s esophagus (BE) surveillance [5]. BE is a metaplastic response to chronic acidic injury in which the normal esophageal squamous cells are replaced by cells more characteristic of the stomach and bowel. The malignant progression of BE to EAC involves a series of histologically characterized stages of change. The risk of progression of BE to EAC, therefore, is dependent on the stage. However, there are known risk factors that promote cancer risk, such as age, tobacco history, and obesity [5,22]. The American College of Gastroenterology recommends one-time endoscopic screening for EAC for patients with chronic GERD symptoms in addition to at least three BE risk factors, which include age greater than 50, White race, male sex, tobacco use, and a first-degree relative with BE or EAC [23]. Continued BE screening to assess disease progression is based on the histologic findings of the prior endoscopic BE diagnosis [5,23].

### 3.2. Diagnostic Modalities

#### 3.2.1. White Light Endoscopy

White light endoscopy has been the standard initial thorough evaluation of BE; however, over the years, advancements in higher acuity endoscopy techniques have led to better detection. For example, chromoendoscopy is a technique in which a dye such as acetic acid solution is used to further enhance lesion identification for EAC [24]. Chromoendoscopy can also be performed using narrow-band imaging, which can better visualize mucosal blood vessels during diagnosis [7,24].

#### 3.2.2. Lugol Chromoendoscopy

Similarly, the pre-malignant process preceding ESCC is squamous dysplasia. Lugol chromoendoscopy is the current gold standard for identifying ESCC and related precursors [25]. Lugol’s iodine is sprayed during the EGD staining normal cells brown due to high glycogen content, but dysplastic or malignant cells remain unstained due to lower glycogen content. Kato et al. reviewed 20 ESCC lesions that underwent ESD and found greater visibility when iodine was added to texture and color enhancement imaging (TXI) compared to the gold standard [25]. Other endoscopic techniques have been added to increase diagnostic accuracy, including confocal laser endomicroscopy (CLE) and high-resolution microendoscopy (HRM). Through enhancing visualization of epithelial cell features and vascular networks, CLE or HRM helps the endoscopist identify and therefore biopsy lesions more likely to be ESCC [26].

#### 3.2.3. Narrow-Band Imaging

Narrow-band imaging (NBI) is an imaging-based biopsy technique that utilizes narrow bands of wavelength, allowing for the identification of abnormal mucosa and vascular patterns with two wavelengths emitted to penetrate varying depths. It is more sensitive for SCC detection compared to white light endoscopy. Of note, Ono et al. performed a study comparing NBI versus Lugol chromoendoscopy in detecting superficial ESCC, in which lesions that were missed by NBI were analyzed. They found that missed lesions tended to be in the anterior wall or were numerous and irregularly shaped. This suggests that while there are advantages to NBI being a preferred modality for ESCC, Lugol chromoendoscopy has usefulness in certain lesions in patients who are at high risk of ESCC or who have had known anterior lesions [27]. In Japan, ultra-magnifying endoscopes offer an optical magnification function up to 520×. This allows for visualization of cellular atypia in real time, including thinner cells and concentrated nuclei after staining, and a more detailed view of blood vessels [28]. However, these techniques are still an emerging area as standardizations of mucosa and microvasculature patterns need to be established for consistency of interpretation [29].

#### 3.2.4. Wide-Area Transepithelial Sampling

The American College of Gastroenterology (ACG) and American Gastroenterology Association (AGA) do not recommend routine BE screening, but instead endorse an individualized approach for screening considerations. Current practice for BE screening sampling is performed by forceps biopsy with the Seattle protocol, by taking random four-quadrant biopsies every 1–2 cm throughout the entire length of the BE segment [30]. However, there is potential risk for missing lesions as only about 5% of the BE segment is sampled, and the ACG has estimated the protocol accuracy anywhere from 35 to 68% [30]. Wide-area transepithelial sampling (WATS) has been developed as an adjunct that utilizes brush biopsy sampling with computer-assisted pathology analysis. There is still limited data regarding the usage of WATS in BE screening, but a meta-analysis by Kumar et al. found that the use of WATS with current forceps biopsy methods improved absolute and relative detection rates in BE than forceps biopsy alone [30].

#### 3.2.5. Multifunctional Ablative Gastrointestinal Imaging Capsule

Regardless of the type of cancer, endoscopy remains vital for visualization and biopsy of the lumen of the esophagus. A recently developed method of screening for conditions such as BE or EC is the multifunctional ablative gastrointestinal imaging capsule (MAGIC) developed by Park et al. This new technology is a pill capsule that combines optical coherence tomography (imaging technology that can provide real-time cross-sectional imaging down to microns) with an ultracompact endoscopic camera and an ablation laser in a pill capsule-sized device [31]. The pill capsule is “tethered”, allowing the proceduralist to control descent and positioning. While not yet validated in human models, efforts are underway to conduct pilot validation studies [31].

#### 3.2.6. Non-Endoscopic Cell-Collection Devices

The Cytosponge collects cytology from a tethered sponge, initially compressed and swallowed in capsule form, retrieving esophageal cells to be assessed for *TFF3*, *p53,* and atypical cells (markers of intestinal metaplasia and dysplasia). In the BEST-3 trial, 131 patients, 2% of 6834 participants in the intervention group (Cytosponge), were diagnosed with BE, 9 patients with dysplastic BE, and 5 patients with stage 1 esophageal cancer, compared to 13, 0, and 0 patients, respectively, in the standard of care group. The Cytosponge, coupled with biomarkers and risk factors, increased the detection of BE and early cancer in clinic patients on treatment for reflux disease compared to standard practice [32]. ACG recognized the need for non-invasive screening tools and endorsed Cytosponge as an option for screening for BE due to its excellent tolerability, safety, and sensitivity [33].

EsophaCap is similar in concept to the Cytosponge but uses a smaller diameter. Its 5 methylated DNA marker panel was validated in a training cohort (*n* = 199) and a testing cohort (*n* = 89), and saw >90% sensitivity and specificity for BE diagnosis in both cohorts. Further studies are underway to validate this data and possibly adjust the markers [34].

EsoCheck is another non-invasive screening tool for BE. This swallowable balloon capsule is attached to a thin catheter and, with its ridges, collects esophageal cells for methylated DNA biomarker analysis (*VIM* and *CCNA1*). It demonstrated a sensitivity and specificity of >90%. Further studies are needed to validate these findings [33,35].

## 4. Role of Endoscopy in Diagnosis and Staging

### 4.1. Diagnosis and Guidelines

Patients with symptoms of new dysphagia, gastrointestinal bleeding, recurrent aspiration or emesis, weight loss, and/or loss of appetite should undergo upper endoscopy evaluation. Esophageal cancer diagnosis is made with adequate tissue sampling (>6 biopsies) for histopathological and molecular assessment. Staging guides treatment decisions; the most accurate tools should be used [36]. Current guidelines recommend initial esophageal cancer staging with computed tomography of the chest and abdomen, and if there is extension below the diaphragm, then additional evaluation of the pelvis. When there is an absence of distant metastases and potential for curative treatment, PET-CT is recommended, followed by endoscopic ultrasound (EUS) for locoregional staging [37].

The 8th edition of the American Joint Committee on Cancer (AJCC) classifies esophagus and esophagogastric junction cancers into three groupings: clinical (cTNM), pathologic (pTNM), and post-neoadjuvant therapy (ypTNM). Clinical grouping is primarily based on imaging with minimal histologic information, distinguishing it from pathologic grouping, which includes review of specimen cell types. As such, pTNM has greater accuracy than cTNM, though it has staging limitations in advanced-stage disease and post-chemotherapy due to destruction and inflammation of nearby structures, thus the creation of post-neoadjuvant staging. TNM categories reflect cancer facts; T refers to the main tumor size and extent, N refers to lymph node involvement, and M refers to metastases as seen in Table A1 [38]. The identified histopathology heavily influences the prognosis and survival profile; for example, early-stage ESCC is worse than early-stage EAC.

### 4.2. Role of EUS in Staging with EUS, Mini-EUS, Contrast EUS

**Figure 1:**
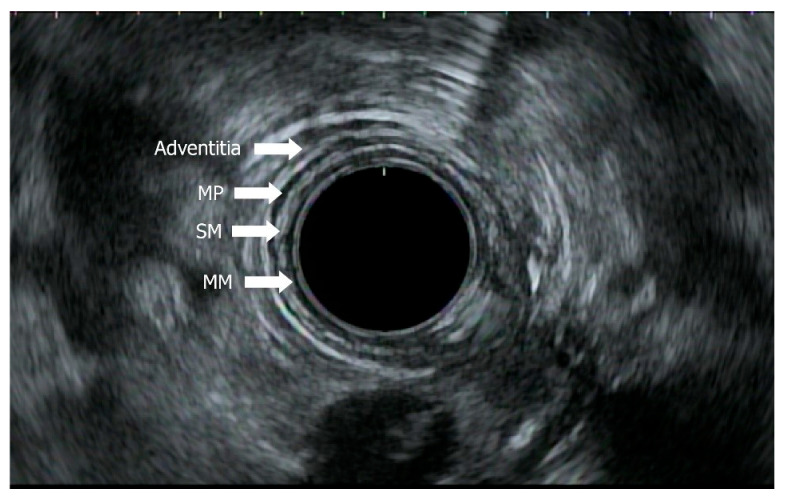
Endoscopic ultrasound of normal esophageal wall layers. MM: mucosa; SM: submucosa; MP: muscularis propria.

EUS is a minimally invasive procedure utilizing high-frequency sound waves to visualize tumor penetration of esophageal wall layers, surrounding tissue, and lymph node involvement. It is the most sensitive test for locoregional staging with varying sensitivity according to TNM stage [39]. Radial echoendoscope, curvilinear echoendoscope, and probe-based EUS are used in staging to provide distinctive views of five esophageal layers: hyperechoic superficial mucosa, hypoechoic deep mucosa, hyperechoic submucosa, hypoechoic muscularis propria, and hyperechoic adventitia, from innermost to outermost layers as seen in Figure 1 [40,41].

The radial echoendoscope, with 7.5 to 12 MHz, provides circumferential staging up to 3 to 5 cm of surrounding tissue. However, if a biopsy or fiducial marker is desired, a curvilinear scope should be used to evaluate structures in the same plane prior to intervention [42]. EUS provides depth of tumor invasion and nodal metastasis. Limitations of EUS include over-staging of nodal involvement; however, accuracy is increased when combined with complementary PET-CT or other imaging.

Identification and evaluation of sentinel lymph nodes has a notable role in guiding treatment and predicting prognosis in EC. Sentinel lymph nodes are typically mapped out using a tracer; however, this is not typically feasible in the cases of EC due to anatomic limitations of tumor location and lymph node accessibility. Therefore, fine needle aspiration (FNA) through EUS for sampling of all potentially suspicious lymph nodes is commonly pursued as standard of care. Other proposed modes of staging include EUS elastography, contrast-enhanced EUS (CH-EUS), and EUS mini-probes. Elastography has two different systems: strain waves, which detect tissue hardness, and shear waves, which measure velocity. Endoscopists may advocate for complementary elastography over sampling when lymphadenopathy is present and sampling is risky due to structures in the trajectory; in addition, elastography indications currently exist for pancreatic and nodal disease [43,44,45]. CH-EUS captures target lesions highlighted by contrast influx and washout period. A pilot study from 2023 showed that contrast-enhanced ultrasound for sentinel lymph node identification can offer greater sensitivity than the traditional EUS-guided approach [46]. Currently, it is primarily used for the diagnosis and treatment of pancreaticobiliary disease and other vascular lesions. According to the Asian Federation of Societies for Ultrasound in Medicine and Biology on CH-EUS, there is no consistent, unified, and reliable enhancement pattern to discern between benign and malignant lymph nodes. Criteria based on heterogenous or homogenous enhancement for malignant or benign nodes, respectively, in four studies resulted in a CH-EUS sensitivity of 83–100% for malignant lymph node diagnosis and 77.3% specificity for benign lymph nodes. The value added of CH-EUS for nodal disease in EC patients with EUS-FNA remains inconclusive at this point [47].

EUS mini-probes (EUS-MPs) are flexible and compact, allowing for manipulation through the working channel of any endoscope. With higher frequencies of 20–30 MHz, it is capable of depicting high-resolution images; however, its purview is limited to roughly 20 mm in depth, primarily capturing intestinal wall lesions and some organs in proximity. The beauty of EUS-MP is its easy-to-use, handy, and adjunct diagnostic tool during unexpected endoscopic findings that necessitate immediate evaluation [5,48,49].

While EUS allows for visualization and sampling of tissue, one of its limitations is over-staging. Since the depth of invasion heavily impacts treatment selection in EC, distinguishing between the degree of layer involvement is vital [50]. In Japan, where ESCC is more prevalent, multiple studies aimed to ascertain the diagnostic value of EUS in diagnosing ESCC. Ishihara et al. found that the addition of EUS did not improve diagnostic accuracy regarding cancer invasion depth in patients with T1 ESCC [50]. They note that the addition of EUS resulted in a 6.6% increase in the proportion of overdiagnosis (thus leading to a potential increase in unnecessary procedures like esophagectomy when cure could be achieved by endoscopic resection), and a 4.5% decrease in the proportion of underdiagnosis (potentially leading to under-treatment with endoscopic resection alone) [50].

## 5. Role of Endoscopy in Treatment

The curative option for EC was previously surgical esophagectomy, removing the cancer segment and 3–4 inches of healthy esophagus and subsequently connecting the remaining esophagus to the stomach in the chest or neck. Though curative, the operative mortality was 2% and there existed high morbidity (bleeding, strictures, leakage, infection, prolonged hospitalization). Endoscopic management of BE, high-grade dysplasia (HGD), and EAC garnered interest over the years and found no difference between endoscopic eradication therapy (EET) and esophagectomy in overall 1-, 3-, and 5-year survival and EAC mortality [23,51]. Although EET saw higher rates of neoplastic recurrence (RR 9.5, 95% CI 3.26–27.75), there was no difference in complete eradication of HGD/EAC, with most achieving complete eradication of intestinal metaplasia within three endoscopic sessions [52,53,54]. Endoluminal therapies are less invasive, more affordable, improve post-operative quality of life, and are a viable option in patients who are not surgical candidates; however, they should be coupled with aggressive surveillance to achieve similar curative rates to prior standard of care.

### 5.1. Resection

Endoscopic mucosal resection (EMR) and endoscopic submucosal dissection (ESD) with ablation are available interventions for BE and are now considered the standard of care [6]. EMR has largely taken the place of esophagectomy for high-grade BE and localized cancer despite the high curative rates of esophagectomy [55]. This is largely in part due to the risks versus benefits of pursuing esophagectomy, which predisposes patients to much higher risks of complications associated with a much more invasive procedure.

The size of the lesion, histology, preoperative endoscopic depth, and risk features often determine which technique to pursue. For ESCC, the ASGE recommends ESD over EMR in early-stage, well-differentiated, nonulcerated cancer lesions > 15 mm, and either EMR or ESD in lesions < 15 mm. For EAC, ESD is recommended over EMR for characteristically similar lesions at a size > 20 mm. Larger, advanced lesions warrant surgical evaluation [56]. For T1 esophageal adenocarcinomas, EMR is pursued for curative resection only in M1-3 lesions and without lymphovascular invasion [57]. This is due to the low risk of nodal involvement and high curative rates seen with this classification and earlier detection. When a tumor involves the submucosa, the risk of nodal involvement and residual disease is increased, and EMR is not advised. Interestingly, in ESCC cases, cautious selection of EMR use begins earlier with M3 lesions compared to S1 lesions in EA. This is evident in a study on the prevalence of metastatic LN in early ESCC, which found nodal metastases in 0%, 0%, 11.8%, 24%, 20/5%, and 43.8%, corresponding to M1, M2, M3, SM1, SM2, and SM3 cancer [58].

Safe and effective EMR is achieved with adjunct modalities that lift the superficial lesion and protect deeper structures. Cap-assisted EMR suctions the mucosa into the cap, allowing for subsequent placement of a band, which allows for safe snare resection [23,58,59]. Injection-assisted EMR results in expansion of the submucosal space followed by snare resection of mucosa [59]. EMR is considered curative if histology demonstrates well-differentiated or moderately differentiated tissues with no lymph node invasion, less than 500 μm submucosal invasion in combination with negative margins [13]. Limitations in EMR lie in submucosal involvement or larger lesions, where resection is piecemeal and of unclear borders; this results in increased risk of disease recurrence.

In considering EMR vs. ESD, the latter has demonstrated greater en bloc resection, curative rates, R0 resection rates, and lower recurrence rates; however, primarily when lesion size was >20 mm [60]. The general ESD technique includes APC to outline the dissecting borders, submucosal injection to elevate the lesion and protect deeper structures, incision with ESD knife according to the APC border, and submucosal plane dissection [24,59,60]. ESD-associated adverse events include bleeding and perforation, with most managed conservatively. Strictures are also a complication of endoscopic resection, with more extensive resections offering greater stricture risk; mucosal defects > 3/4th of the luminal circumference carried a strong risk factor for stricture development, frequently requiring multiple endoscopic balloon dilations [61]. Hanoaka et al. and Hashimoto et al. evaluated the efficacy of steroid injection on post-endoscopic strictures; they demonstrated a significantly lower stricture occurrence rate and fewer endoscopic balloon dilations in the single steroid injection group [62,63]. The JGES guidelines weakly recommend local steroid injection for such extensive lesions, and additional randomized trials are underway [64].

In Japan, endoscopic resection of lesions has favorable short- and long-term outcomes. Magnifying endoscopy is often employed in Japan to enhance the assessment of cancer invasion depth within the mucosal layer with an accuracy of 92.4% [65]. For the pT1a-EP/LPM cohort, recurrence was unlikely even without additional treatment. According to seven studies, for the T1a-MM/T1b-SM1 cohort, lymphovascular invasion ranged from 8.7 to 25.9% with 5-year overall and disease-specific survival rates of 57.3–95.6% and 96.9–98.0%, respectively [66,67,68,69,70,71,72]. Mortality and prognosis were more influenced by additional malignancy, functional and nutritional status, depth of invasion, and age [67,70]. ESGE and JGES guidelines state en bloc R0 resection for well-differentiated ESCC invading T1a-MM/T1b-SM1, without lymphovascular invasion, is curative in most cases [64]. Further treatment should consider the risk of treatment versus nodal metastasis. In Japan, additional treatment is recommended for pT1b-SM ESCC; however, such pursuit for T1a-MM ESCC is still not clearly defined and an area for further discussion [64]. For pT1b or lymphovascular invasion, current guidelines recommend chemoradiotherapy or esophagectomy; however, additional studies are needed to elucidate which treatment better combats adverse events, local and nodal recurrence, and overall and disease-specific outcomes.

### 5.2. Ablative Techniques

Resection of lesions via endoscopic mucosal resection (EMR) or endoscopic submucosal dissection (ESD) is usually followed by endoscopic ablation of residual lesions to prevent further metaplasia and reduce the risk of recurrence [6]. This guideline comes from continued evidence in randomized trials of additional ablation with argon plasma coagulation (APC) versus surveillance, demonstrating significantly higher rates of dysplasia in the surveillance arm [73]. A separate randomized trial comparing radiofrequency ablation (RFA) versus stepwise radical EMR of residual BE lesions conclusively demonstrated sooner complete eradication of intestinal metaplasia (CE-IM) in the RFA arm and with fewer procedures and complications [74]. The high rate of recurrence in untreated segments is thought to be due to the field effect, in which the adjacent residual BE lesions still harbor the same mutated genetic potential because no intervention has led that lesion to revert to normal squamous epithelium [59].

Ablation therapy uses thermal energy that induces coagulation of proteins and cell death. The ablation depth is shallow at 0.5 mm and targeted, allowing for controlled ablation [24]. With this limited and superficial depth from the ablative technique, resection of nodular tissue likely harboring invasive cancer below should be completed prior to ablation to increase the likelihood of CE-IM [24,59]. There are multiple forms of ablation, including radiofrequency, argon plasma coagulation, photodynamic therapy, and cryotherapy, discussed further below.

#### 5.2.1. Radiofrequency Ablation

In RFA, electrodes are displayed over the surface area of the device and deliver high-frequency energy to the contacted tissue. RFA catheters come in all shapes and sizes, ranging from a few millimeters to 4 cm in circumference, allowing for an array of therapeutic options [59]. With its ease of use and effective track record, it is the most used ablation technique in the United States.

#### 5.2.2. Photodynamic Therapy

Another ablative technique includes photodynamic therapy of BE with high-grade dysplasia (HGD). It involves intravenous administration of medication to induce light sensitivity in targeted gastrointestinal tissue, thereby causing tissue injury when light is delivered. The depth and degree of destruction heavily depend on the medication agent and light wavelength delivered, but prior randomized control trials have demonstrated complete remission of dysplasia [75,76]. However, the degree of remission and side effects varies due to ill-defined treatment guidelines secondary to unmeasurable tissue sensitivity, varying wavelength, and treatment duration, and unclear dosage and duration. For these reasons, photodynamic therapy is a less popular ablative technique.

#### 5.2.3. Argon Plasma Coagulation Therapy

APC is a noncontact technique with targeted argon gas delivery and thermal destruction of tissue. Though it demonstrated effectiveness in achieving remission, its use was limited by the risk of perforation and stricture formation. As a result, it evolved to a hybrid-APC wherein submucosal saline injection creates a protective barrier for deeper submucosal structures while providing ample space for adequate ablation and continued higher power settings [77]. Retrospective studies and case series have demonstrated effective complete remission of intestinal metaplasia and lower stricture rate; however, RCTs are necessary to compare the safety and efficacy of hybrid-APC [77].

#### 5.2.4. Cryotherapy

Cryotherapy can be used for both pre-malignant and malignant esophageal conditions. It utilizes liquid nitrogen sprayed through an open-tip catheter to freeze lesions within the esophagus. The concerning lesion is “frozen” by liquid nitrogen. After multiple cycles of freezing and thawing, the lesion is destroyed [78]. A meta-analysis by Chandan et al. found that rates of post-procedural strictures and perforations are similar between cryotherapy and radiofrequency ablation [78]. Cryotherapy is an emerging treatment choice for EAC in patients with stage T1 disease and low risk for nodal metastasis [79]. Standard therapy involves EMR of any visible lesions followed by cryotherapy for any residual Barrett’s esophagus tissue. Tsai et al. followed eighty-eight patients with EAC, who underwent treatment with cryotherapy, until complete local eradication of intraluminal tumor or progression of disease. Eighty-six of the patients completed treatment with a complete response (66.2% for all T1, 76.3% for T1a, 45.8% for T1b, and 6.7% for T2). Adverse events included abdominal pain, dysphagia, sore throat, and chest pain [79,80].

Cryotherapy is a promising palliative treatment of dysphagia in patients with inoperable disease [81]. Kachaamy et al. investigated liquid nitrogen cryotherapy as a means for palliation of dysphagia in inoperable EC; they graded dysphagia of forty-nine patients using the Likert scale: 0 for no dysphagia, 1 for dysphagia to solids, 2 for dysphagia to semisolids, 3 for dysphagia to liquids, and 4 for dysphagia to saliva. The pre-intervention dysphagia score mean was 2.4, and the post-intervention mean was 1.7 [81].

The data on treating ESCC with cryotherapy is very limited. Cash et al. published a case report on a patient with ESCC who underwent cryotherapy for palliative purposes three years after initial chemoradiation; one month after cryotherapy, the tumor was endoscopically in remission but remained present on biopsy. The patient underwent repeat cryotherapy and had complete eradication of dysplasia; biopsies 24 months later remained negative [82]. Further investigation is warranted to discover if cryotherapy could be a treatment option for those with ESCC.

## 6. Stents for the Management of Luminal Occlusion in Esophageal Cancer

Most esophageal cancers are diagnosed when they are already in an advanced stage, often when the esophagus is occluded by the tumor [83]. To relieve the occlusion, there are a variety of stents available; we will focus on self-expanding metal and plastic stents and drug-eluting stents.

Self-expanding metal stents (SEMSs) are some of the most widely used for malignant esophageal obstruction; there are three types: covered, partially covered, and uncovered. Uncovered metal stents have the common complication of regrowth (17–36%) of the malignant tissue through the stent, which could lead to a new stricture or occlusion [83,84]. To combat the growth of tissue causing re-occlusion, there have been a variety of stents produced with an exterior coating to prevent ingrowth. With new innovations come new problems, and SEMSs with coverings often migrate from their original site. This is the most common complication at 36.3% [85]. A SEMS was developed to try and decrease migration. This stent had proximal and distal ends that were uncovered to allow tissue growth through to anchor the stent to the wall of the esophagus, with the central portion of the stent covered to keep malignant tissue from growing through [83]. Additional strategies include over-the-scope and through-the-scope clips to further secure the stent to the esophageal wall [83].

Self-expanding plastic stents (SEPSs) were developed after SEMS and have the advantages of being more comfortable for patients, easier removal, as well as reduced risk of tissue trauma (bleeding, fistulas, perforations, tracheal–bronchial fistulas) [83]. SEPSs increase the quality of life in patients with benign esophageal strictures experiencing dysphagia and are more cost-efficient when compared to two or more dilations [86]. The downside of SEPSs is that there is a much higher risk of stent migration. Holm et al. found that migration was more common when stents were placed for benign strictures (81.8%), followed by anastomotic strictures (75%). Migration was less common in SPES placed for radiation-induced strictures (28.6%). In terms of the location of stent placement, distally and proximally placed SPES were the most likely to migrate (70.4% and 68.1%, respectively) while SPES placed in the mid-esophagus were the least likely to migrate (30%) [87].

Drug-eluting stents (DESs) are an exciting frontier for GI cancer therapy. These stents provide mechanical support for the integrity of the esophagus and release targeted anti-cancer therapy to the site of treatment. No drug-eluting stent is currently available in the United States for the treatment of esophageal cancers, as research for a non-vascular application of DESs is unfortunately slow [83].

## 7. Post-Treatment Surveillance

After endoscopic eradication therapy, patients are typically stratified into different post-treatment surveillance groups due to higher rates of recurrence. Firstly, patients who have Barrett’s esophagus with high-grade dysplasia should undergo upper endoscopy every 6 months for 2 years, then yearly [88]. Secondly, patients who had T1a EAC should undergo endoscopic ultrasonography every 6 months for two years, followed by yearly CT chest/abdomen scans for 5 years [88]. Lastly, patients who had higher-risk resections should undergo endoscopic ultrasound more frequently: every 3 months for the first year post-resection, then every 6 months for the second year, and then yearly thereafter. CT chest/abdomen scans are recommended in shorter intervals as well, starting with every 6 months for the first year, and then yearly for the next 5 years [88].

## 8. Future Directions

Over the past few decades, the role of endoscopy in esophageal cancer has evolved from being a means for tissue sampling to screening, diagnostics, staging, and disease management. Further, techniques such as chromoendoscopy, CLE, HRM, and EUS provided higher acuity approaches promoting earlier detection and enhanced prognostic outcomes. Recently, strategies in targeted screening, individualized surveillance, and treatment have been created and tested to better identify those at high risk for BE transformation. Precision medicine involves genetics, non-invasive studies, and biomarkers to achieve earlier intervention in high-risk individuals. The aim is to balance the low likelihood of dysplastic transformation against resource-intensive and invasive surveillance while knowing the effectiveness of early intervention in a high-mortality disease.

AGA published a best practice advisory in December 2022 titled New Technology and Innovation for Surveillance and Screening in Barrett’s Esophagus. Best Practice Advice 1 recommended changes to screening criteria, removing the mandatory prerequisite of chronic GERD symptoms to qualify for screening [33]. This was supported by multiple studies demonstrating that many patients diagnosed with EAC would not have met current screening criteria, primarily due to a lack of symptomatic GERD. The AGA now advises consideration of screening with standard upper endoscopy in individuals with at least three established risk factors for BE and EAC: white male, age > 50, history of smoking, GERD, obesity, or family history of BE or EAC [33]. The aim is to better capture the at-risk population and detect BE earlier. Widespread acceptance and future studies are needed to validate this approach. We anticipate earlier detection and treatment; however, it would be prudent to also note the possible financial implications, adverse events of invasive procedures, impact on surveillance protocols, increase in outpatient procedural volume, and more.

The second AGA best practice advisory stated that non-endoscopic cell-collection devices can be considered as an option to screen for BE and included current devices: Cytosponge, EsoCheck, and EsophaCap, detailed above. These devices were tolerated well and had excellent sensitivity for the diagnosis of BE in multicenter international studies [33]. Similar to best practice advisory 1, further data and guidance are needed to validate the use of these devices in the US.

Recently, there has been growing interest in non-invasive approaches to BE and esophageal cancer screening. The progression of BE to EAC is typically seen in dysplastic BE cases through a progression of gene mutations that have been an area of interest for identifying biomarkers in both BE and EAC [89]. Genome studies have been performed to identify possible genomic variants conferring increased genetic susceptibility to BE [89]. One group developed a risk score utilizing genetic, clinical, and demographic data [90]. Investigation of genomic DNA biomarkers, such as *TP53* mutations, has been reported in studies to help screen and assess risk stratification in BE patients for progression to EAC [89]. In addition to these non-invasive cell-collection devices and screening tools, serological markers for detection are on the rise. The EMERALD (Oesophageal MicroRNAs of Barrett, Adenocarcinoma, and Dysplasia) study aimed to develop and test a blood-based assay for EAC and BE. They identified six microRNAs that were overexpressed in the sera of diseased patients compared to non-diseased patients and subsequently trained a machine learning model. This novel blood test identified affected individuals with an AUROC of 97.6% in the training cohort and 91.9% in the validation cohort [91]. Further studies are needed to validate and guide the use of these liquid biopsies as a complement in BE/EAC screening.

Standard guidelines are used for surveillance endoscopy of non-dysplastic and dysplastic BE, i.e., 3–5 years for non-dysplastic BE and 3–12 months for indefinite or low-grade dysplasia. By utilizing these non-invasive tools created on the principle that certain genomic alterations are strong indicators of BE transformation, surveillance endoscopy may be tailored to the individual’s true risk of EA, thereby balancing excessive low-risk endoscopies with early detection of high-risk lesions. The landscape of non-invasive modalities for BE and EAC detection is evolving and a necessity. Ongoing trials are underway to validate these promising tools. Full adoption of this practice will be contingent on further validation of biomarkers, creation of standard guidelines, cost-effectiveness analysis, more comparative trials, and societal endorsements. In addition to surveillance and diagnostics, additional studies regarding treatment and adverse effects, such as management of pT1a-MM lesions and post-resection strictures with steroid use, need to be further investigated and protocolized.

The potential role and use of artificial intelligence (AI) has been an emerging topic in medicine. There have been some studies investigating the utility of AI learning systems in diagnosing esophageal cancers. Nakao et al. recently conducted the first prospective RCT to our knowledge on how the use of AI in detecting ESCC in a clinical setting. Patients who were deemed high risk for ESCC and planned for EGD were randomly assigned to either the control group, in which the endoscopist performed the EGD using a normal monitor, or the treatment group, in which the endoscopist also used an AI monitor that helped to detect ESCC with annotations in addition to the normal monitor. The study found that there was no significant improvement in cancer detection when the AI support system was used by the endoscopists [92].

With a guarded 5-year prognosis, EC remains a challenging and prevalent malignancy worldwide. Endoscopy has become a pivotal tool in many aspects of EC that offers the potential for improved outcomes for patients in the future. The rise in new technology and innovation, from screening to surveillance to treatment, enhances the endoscopic role in early detection and treatment, thus improving this malady.

## Data Availability

Not applicable.

## Abbreviations

The following abbreviations are used in this manuscript, in order of appearance:

EC	Esophageal cancer
AGA	American Gastroenterological Association
AJCC	American Joint Committee on Cancer
APC	Argon plasma coagulation
ASGE	American Society of Gastrointestinal Endoscopy
ASIR	Age-standardized incident rates
ASMR	Age-standardized mortality rates
BE	Barrett’s esophagus
CE-IM	Complete eradication of intestinal metaplasia
CH-EUS	Contrast-enhanced endoscopic ultrasound
CLE	Confocal laser endoscopy
DESs	Drug-eluting stents
EAC	Esophageal adenocarcinoma
EET	Endoscopic eradication therapy
EMERALD	Oesophageal MicroRNAs of Barrett Adenocarcinoma and Dysplasia
EMR	Endoscopic mucosal resection
EOE	Eosinophilic esophagitis
ESD	Endoscopic submucosal dissection
GERD	Gastroesophageal reflux disease
EUS	Endoscopic ultrasound
EUS-MPs	Endoscopic ultrasound mini probes
FNA	Fine needle aspiration
NSAIDs	Non-steroidal anti-inflammatory drugs
GIST	Gastrointestinal stromal tumor
HGD	High-grade dysplasia
HRM	High-resolution microendoscopy
MAGIC	Multifunctional ablative gastrointestinal imaging capsule
NBI	Narrow band imaging
SEPSs	Self-expanding plastic stents
WATS	Wide-area transepithelial sampling

## Appendix A

**Table A1:** A TNM classification system.

Category	Criteria
T category	
TX	Tumor cannot be assessed
T0	No evidence of primary tumor
Tis	High-grade dysplasia, malignant cells confined by basement membrane
T1	Tumor invades lamina propria, muscularis mucosae, or submucosa
T1a	Tumor invades lamina propria or muscularis mucosae
T1b	Tumor invades submucosa
T2	Tumor invades muscularis propria
T3	Tumor invades adventitia
T4	Tumor invades adjacent structures
T4a	Tumor invades the pleura, pericardium, azygos vein, diaphragm, or peritoneum
T4b	Tumor invades other adjacent structures, such as aorta, vertebral body, or trachea
N category	
NX	Regional lymph nodes cannot be assessed
N0	No regional lymph node metastasis
N1	Metastasis in 1–2 regional lymph nodes
N2	Metastasis in 3–6 regional lymph nodes
N3	Metastasis in 7+ regional lymph nodes
M category	
M0	No distant metastasis
M1	Distant metastasis
